# Beneficial Effects of Hordenine on a Model of Ulcerative Colitis

**DOI:** 10.3390/molecules28062834

**Published:** 2023-03-21

**Authors:** Zhengguang Xu, Qilian Zhang, Ce Ding, Feifei Wen, Fang Sun, Yanzhan Liu, Chunxue Tao, Jing Yao

**Affiliations:** 1 School of Basic Medicine, Jining Medical University, Jining 272067, China; 2 School of Basic Medicine, Weifang Medical University, Weifang 261000, China; 3 Jining Key Laboratory of Pharmacology, Jining Medical University, Jining 272067, China

**Keywords:** hordenine, ulcerative colitis, SPHK-1/S1PR1/STAT3 signaling, Rac1

## Abstract

Hordenine, a phenethylamine alkaloid, is found in a variety of plants and exhibits a broad array of biological activities and pharmacological properties, including anti-inflammatory and anti-fibrotic effects. However, the efficacy and underlying mechanisms of hordenine in treating ulcerative colitis (UC) remain unclear. To address this, we examined the therapeutic effects of hordenine on dextran sodium sulphate (DSS)-induced UC by comparing disease activity index (DAI), colon length, secretion of inflammatory factors, and degree of colonic histological lesions across diseased mice that were and were not treated with hordenine. We found that hordenine significantly reduced DAI and levels of pro-inflammatory factors, including interleukin (IL)-6, IL-1β, and tumor necrosis factor alpha (TNF-α), and also alleviated colon tissue oedema, colonic lesions, inflammatory cells infiltration and decreased the number of goblet cells. Moreover, in vitro experiments showed that hordenine protected intestinal epithelial barrier function by increasing the expression of tight junction proteins including ZO-1 and occludin, while also promoting the healing of intestinal mucosa. Using immunohistochemistry and western blotting, we demonstrated that hordenine reduced the expression of sphingosine kinase 1 (SPHK1), sphingosine-1-phosphate receptor 1 (S1PR1), and ras-related C3 botulinum toxin substrate 1 (Rac1), and it inhibited the expression of phosphorylated signal transducer and activator of transcription 3 (p-STAT3) in colon tissues. Thus, hordenine appears to be effective in UC treatment owing to pharmacological mechanisms that favor mucosal healing and the inhibition of SPHK-1/S1PR1/STAT3 signaling.

## 1. Introduction

Ulcerative colitis (UC) is a multifactorial chronic inflammatory bowel disease (IBD), which mainly affects the colon and rectum. Clinical manifestations in patients include abdominal pain, diarrhea, and bloody stool [1,2]. However, the etiology and pathogenesis of UC remain somewhat unclear and may be related to genetic factors, environmental factors, infections, dysbiosis, and immune factors [2,3,4,5,6,7]. At present, drug treatments for UC include 5-aminosalicylic acid (5-ASA), glucocorticoids, immunosuppressants, and biological agents. Unfortunately, the effects of most of these drugs are limited in terms of therapeutic efficacy and safety, resulting in various adverse reactions [3,8,9]. Therefore, safer and more effective drugs for UC treatment are urgently required.

The sphingolipid pathway is a key component of many signal transduction pathways and regulates biological activities such as cell proliferation, cell migration, and inflammation [10,11]. Sphingomiglide kinase-1 (SPHK1) is an intracellular lipase that acts as a signaling molecule in the sphingolipid signaling pathway, converting sphingosine into Sphingosine-1-phosphate (S1P) [12,13,14]. S1P is a pleiotropic bioactive sphingolipid metabolite that regulates various cellular processes by binding to its own receptors [15,16,17]. Studies have shown that S1P is closely related to the occurrence and development of UC [17,18]. Sphingosine-1-phosphate receptor (S1PR1), also known as endothelial differentiation gene 1 (EDG1), is a G-protein-coupled receptor that mediates the bioactivity of S1P to promote cell proliferation and survival [19]. The activation of the SphK1-S1P-S1PR1 axis is crucial for various cellular signaling cascades and several pathological processes [20]. Signal transducer and activator of transcription 3 (STAT3) mediates cytokine and growth factor signals in a range of tissues and translocates to the nucleus after phosphorylation, where it participates in the regulation of cell proliferation, migration, differentiation, and transcription of inflammatory target genes [21,22]. Numerous studies have demonstrated that epithelial STAT3 is essential for maintaining gut barrier integrity [23,24], whereas S1P promotes the activation of STAT3, thus contributing to the development of colitis-related cancers [17].

**Figure 1:**
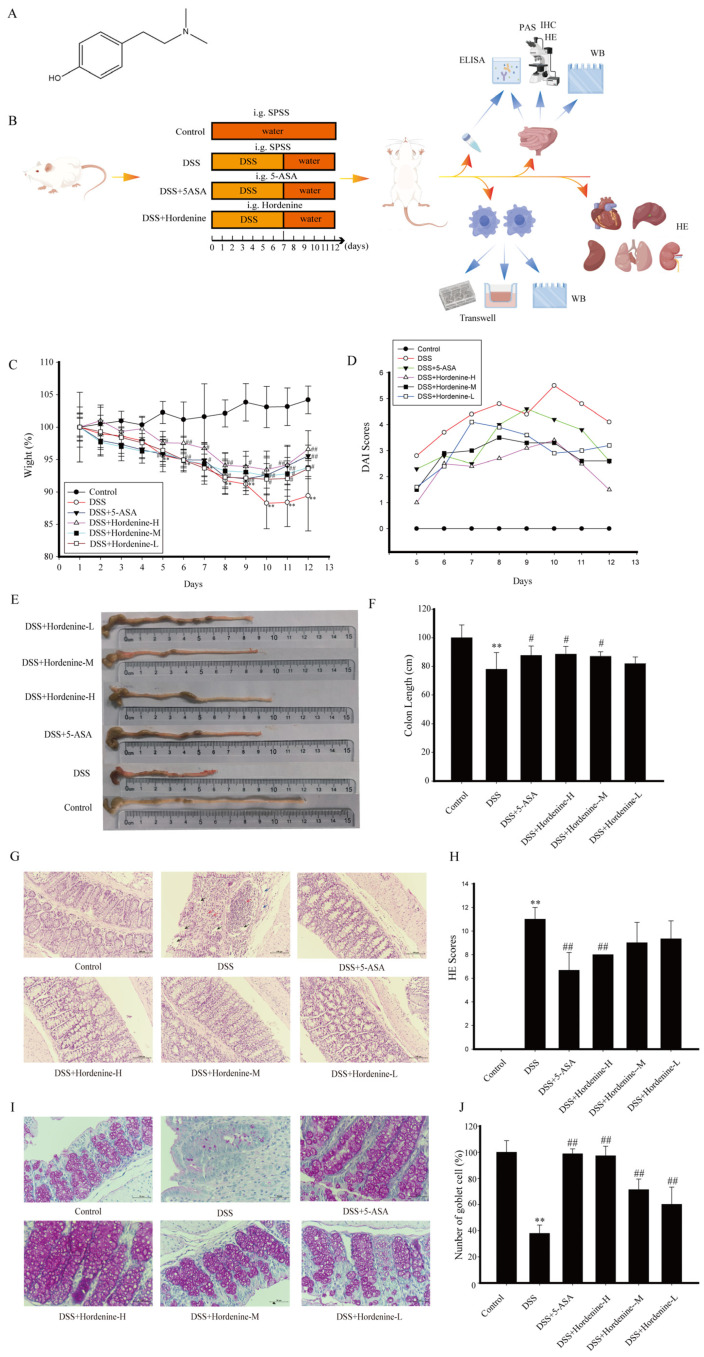
Hordenine ameliorated DSS-induced UC in mice. (**A**) The chemical structure of hordenine. (**B**) Flow chart of the experimental design. SPSS, as known as stroke-physiological saline solution; ELISA: enzyme linked immunosorbent; HE: hematoxylin-eosin staining; IHC: immunohistochemistry; WB: western blot; PAS: periodic acid-Schiff (PAS) staining; the elements in the graph are derived from figdrow. (**C**) Changes in body weight. ** *p* < 0.01 vs. control group; ^#^ *p* < 0.05, ^##^ *p* < 0.01 vs. DSS group. (**D**) The DAI scores were calculated. (**E**) The macroscopic representations of colons. (**F**) The colon lengths among the six groups. ** *p* < 0.01 vs. control group; ^#^ *p* < 0.01 vs. DSS group. (**G**) Hematoxylin and eosin staining of colonic sections. Ulcer and necrosis are indicated by black arrow. Inflammatory cell infiltration and aggregation are indicated by red arrow. Tissue edema is indicated by blue arrow. Scale bar: 100 μm. (**H**) Histological changes. ** *p* < 0.05 compared with control group; ^##^ *p* < 0.05 compared with DSS group. (**I**) periodic acid-Schiff staining of colonic sections. (**J**) Goblet cells count. ** *p* < 0.05 compared with control group; ^##^ *p* < 0.05 compared with DSS group.

Hordenine, chemically known as 4-(2-Dimethylaminoethyl) phenol, is an alkaloid extracted from a wide range of plants, including cacti and the seedlings of cereals (such as barley, proso millet, and sorghum), as well as some algae and fungi. It exhibits antioxidant, anti-inflammatory, antibacterial, and anti-tumor activities [25,26,27,28,29,30]. The chemical formula for hordenine is given in Figure 1A. Studies indicate that hordenine can inhibit neuro-inflammation and reduce nerve pain [27], exert anti-inflammatory effects in diabetic nephropathy [30], and prevent lipopolysaccharide-induced acute lung injury [25,27]. In addition, hordenine displays antibacterial properties, as well as antiviral properties against herpes virus and SARS-CoV-2, and prevents inflammation caused by both viruses at the site of infection [31,32]. However, the potential alleviating effects of hordenine on experimentally-induced UC and the underlying mechanisms driving these effects remain unclear. Here, we addressed this gap using a dextran sodium sulphate (DSS)-induced UC model and in vitro experiments to provide a theoretical basis for the use of hordenine in the treatment of UC.

## 2. Materials and Methods

### 2.1. Ethics Statement

All animal experiments were approved by the Institutional Animal Care and Use Committee of Jining Medical University (2021-DW-ZR-019).

### 2.2. Chemicals and Reagents

Hordenine (high-performance liquid chromatography-grade ≥95%) and 5-ASA (purity > 99%) were purchased from Shanghai Yuanye Bio-Technology Co., Ltd. (Shanghai, China). DSS (molecular weight 36~50 kDa) was purchased from MP Biomedicals (Irvine, CA, USA). Fecal occult blood qualitative detection kit purchased from Shanghai Yuanye Bio-Technology Co., Ltd. (Shanghai, China). Mouse interleukin (IL)-6, IL-1β, and tumor necrosis factor-alpha (TNF-α) enzyme-linked immunosorbent assay (ELISA) kits were purchased from Biolegend (San Diego, CA, USA). Hematoxylin and eosin (H&E) were purchased from Solarbio Science & Technology Co., Ltd. (Beijing, China). Periodic acid-Schiff (PAS) staining solution was purchased from Wuhan Servicebio technology Co., Ltd. (Wuhan, China). Universal SP Kit was purchased from ZSGB-BIO Co., Ltd. (Beijing, China). Primary antibodies against SPHK1, S1PR1/EDG1, ras-related C3 botulinum toxin substrate 1 (Rac1), and p-STAT3 (phospho-S727) were purchased from Abcam (Cambridge, MA, USA). Antibodies against IL-6, IL-1β, and TNF-α were purchased from Bioworld Technology (St. Louis Park, MN, USA). Antibodies against β-actin and zonula occludens 1 (ZO-1) were purchased from Affinity Biosciences (Cincinnati, OH, USA). Antibodies against occludin was purchased from Proteintech Group (Wuhan, China). Anti-mouse and anti-rabbit secondary antibodies were obtained from eBioscience (San Diego, CA, USA). Enhanced chemiluminescent (ECL) substrate was purchased from Beijing Labgic Technology Co., Ltd. (Beijing, China). Transwell inserts (pore size of 0.4 µm) were purchased from Corning Inc. (Kennebunk, ME, USA).

### 2.3. Cell Culture

Mouse colonic epithelial cells (MCECs) were cultured in Dulbecco’s modified Eagle’s medium (DMEM) supplemented with 10% fetal bovine serum (FBS), 100 U/mL penicillin, and 100 μg/mL streptomycin in an incubator with 5% CO_2_.

### 2.4. Cell Counting Kit-8 (CCK-8) Experiment

MCEC cells at the logarithmic growth stage were evenly distributed in 96-well plates at a growth density of 30%, and after 24 h of incubation, a blank group (no cells were inoculated), a control group, and various concentrations of hordenine administration groups (500, 250, 125, 62.5, 31,25, and 15.625 μM) were set up, with six replicate wells in each group. Each well was given the medication for 24 h before the addition of 10 μL CCK-8 reagent and an additional hour of incubation. The aforementioned test was carried out three times [33]. It was found that 500, 250, 125, 62.5, 31.25, and 15.625 μM of hordenine had no significant effect on the survival rate of MCEC cells for 24 h. Therefore, 500 μM was chosen as the hordenine administration condition.

### 2.5. DSS-Induced Models and Hordenine Treatment

Female BALB/c mice (35~40 days, weighing 18~22 g) were obtained from Jinan Pengyue Experimental Animal Breeding Co., Ltd. (Jinan, China). Animals had access to food and water ad libitum and were maintained on a 12 h/12 h light/dark cycle at 21 ± 2 °C with a relative humidity of 45 ± 10%.

All mice were then randomly divided into six groups (*n* = 10/group): untreated control, DSS model, DSS+5-ASA 40 mg/kg, and DSS+hordenine 50 mg/kg, DSS+hordenine 25 mg/kg, DSS+hordenine 12.5 mg/kg (Figure 1B). Except for the control group, mice were given 4% (*w*/*v*) DSS solution for 7 days before being given regular water for the next 5 days [33]. From day 1 to day 12, mice in the treatment groups were administered 5-ASA or hordenine daily by gavage, while mice in control and DSS model groups were administered normal saline. All mice were sacrificed on day 13.

### 2.6. Assessment of Disease Activity Index (DAI) Score

From the first day of the experiment, mice were observed daily for their mental status and hair condition. Their body weight, fecal characteristics and fecal occult blood were recorded and DAI scores were calculated [17]. Specific standards of DAI score are shown in Table 1.

### 2.7. Collection of the Main Organs and Colon Tissues and Preparation of Serum Samples

The mice were sacrificed on 13th day, and the main organs including heart, liver, spleen, lung and kidney, were collected and weighed, and the colon and rectum were separated from the small intestine at the proximal ileocecal end, and from the anus at the distal end. This section of colorectal was extracted and straightened (without stretching) and its length was measured with a ruler, and follow-up tests were performed [34].

The colons were dissected longitudinally and washed with saline, and fixed with 10% neutral formalin for H&E staining, PAS staining and immunohistochemical (IHC) staining. The remaining colon tissue was stored at −80 °C for Western blot analysis.

### 2.8. H&E Stain

Paraffin-embedded colonic tissue blocks were cut into 4 μm-thick sections for H&E staining [35]. The histological change scoring criteria are shown in Table 2.

### 2.9. PAS Stain

PAS staining is used to display glycogen and polysaccharide substances, mostly used for goblet cell staining. The paraffin tissue section was dewaxed and hydrated, and then stained with PAS staining solution B, A and C according to the instructions, and finally dehydrated and sealed, and the image was collected and analyzed under the microscope. The experimental method is consistent with the literature report [36].

### 2.10. Measurement of Cytokines

The levels of IL-6, IL-1β, and TNF-α in the supernatant of colon tissue homogenate were determined by ELISA according the instruction of test kits [37].

### 2.11. IHC

Colon segments were taken and fixed in 4% paraformaldehyde. Then, 4 μm-thick paraffin-embedded colon sections were prepared and incubated in 0.3% hydrogen peroxide methanol for 20 min at room temperature to inhibit endogenous peroxidase activity. For antigen extraction, sections were treated with citrate buffer (pH 6.0) and heated three times in a microwave oven for 5 min each. Sections were then closed with 5% bovine serum albumin (BSA) for 30 min at room temperature, followed by overnight incubation at 4 °C with the IL-6, TNF-α, SPHK1, S1PR1, Rac1 and p-STAT3 primary antibody. The next day, the sections were incubated with their respective pairs with secondary antibodies at room temperature, and the remaining manipulations were completed according to the Universal SP kit, and finally observed and photographed under a microscope [38,39].

### 2.12. Western Blot Assay

The BCA assay reagent was used to measure the protein concentration after removing the whole protein from the colon tissue of the mice in each group. After electrophoresizing the samples from each group in polyacrylamide gels of 8%, 10%, and 15%, the protein was then transferred to a polyvinylidene fluoride membrane. The membrane was blocked with 5% skim milk powder for 2 h at room temperature and then incubated with primary antibody overnight at 4 °C. After that, the membranes were exposed to secondary antibodies for 1 h at room temperature. As directed by the ECL western blotting experiment, membrane imaging was carried out. The protein expression of IL-6, TNF-α, IL-1β, SPHK1, S1PR1, Rac1 and p-STAT3 in colon tissues, and ZO-1 and occludin in MCECs were examined using the previously described method [38,39].

### 2.13. Co-Culture and Scratch Assay

Colitis model mice were induced with 4% DSS solution for 5 days and sacrificed on the 6th day. Peritoneal macrophages (Mφs) were collected and cultured in DMEM. MCECs were plated in 6-well culture plates and incubated at 37 °C in a 5% CO_2_ incubator. Mφs were added to the upper chamber of a Transwell insert (pore size of 0.4 μm) on the cavity, co-cultured with MCECs in 6-well culture plate, and treated with hordenine (500 μM). Following the above treatments, monolayers of the MCECs were scratched and observed at 0 and 24 h. The percentage of coverage was calculated.

### 2.14. Statistical Analysis

Statistical analysis was performed using Prism 5 software (GraphPad Software, La Jolla, CA, USA). All data were presented as the mean ± SD. The significant difference between groups were analyzed by Student’s *t*-test (for normal distribution), *p* values of less than 0.05 were considered significant for all data.

## 3. Results

### 3.1. Hordenine Ameliorates DSS-Induced UC in Mice

Mice in the DSS group displayed a significant decrease in body weight compared to those in the control group, but this DSS-induced weight loss was alleviated by treatment with hordenine (Figure 1C). Average DAI scores were higher among mice in the DSS group compared to those in the control group (Figure 1D). The drugs did not affect the weight of the heart, spleen, lungs, kidneys, and liver weight. The colon lengths of mice in the model group were significantly shorter than those of mice in the control group, and were significantly longer among mice in the drug administration group (Figure 1E,F). Our examination of colon injury and inflammatory cell infiltration using HE staining. These results revealed colonic necrosis and ulceration, disappearance of mucosa and glands, hyperplasia of connective tissue, granulocyte infiltration, and infiltration of a large number of inflammatory cells in mice in the DSS group, all of which were substantially improved by treatment with hordenine (Figure 1G,H). In addition, PAS staining revealed that compared with the normal group mice, the goblet cells of the model group mice were irregular in arrangement, incomplete in shape, significantly reduced in number and uneven in size, some cells were vacuolate, and the contents of glycogen and other mucus substances were significantly reduced. Compared with the model group, the goblet cells in the hordenine group were more regular in arrangement, more complete in shape, and significantly increased in number, and contained more mucus substances such as glycogen, and showed a certain dose dependence (Figure 1I,J).

### 3.2. Hordenine Inhibited the Secretion and the Expression of Inflammatory Factors Induced by DSS

**Figure 2:**
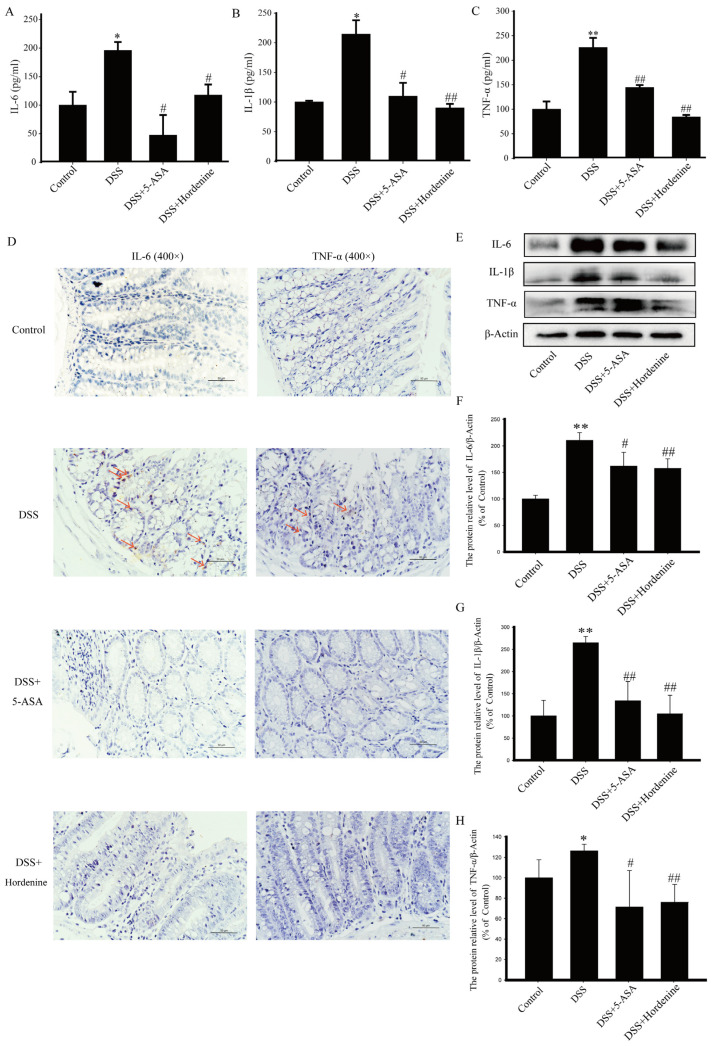
Hordenine (50 mg/kg) reduces levels of inflammatory mediators in the colon of mice with dextran sodium sulfate (DSS)-induced colitis. Enzyme-linked immunosorbent analysis of IL-6 (**A**), IL-1β (**B**) and TNF-α (**C**) in colon tissue supernatants. * *p* < 0.05, ** *p* < 0.01 vs. control group; ^#^ *p* < 0.05, ^##^ *p* < 0.01 vs. DSS group. (**D**) Immunohistochemical staining of IL-6 and TNF-α in colon tissues.Red arrows point to the positive expression of the target protein, scale bar: 50 μm. (**E**–**H**) Western blotting analysis of IL-6, IL-1β and TNF-α expression in colon tissues. * *p* < 0.05, ** *p* < 0.01 vs. control group; ^#^ *p* < 0.05, ^##^ *p* < 0.01 vs. DSS group.

As shown in Figure 2A–C, hordenine inhibited DSS-induced production of interleukin (IL)-6, IL-1β, and tumor necrosis factor alpha (TNF-α) in the supernatant of colon tissue homogenate. The results of immunohistochemistry (IHC) and western blotting showed that hordenine inhibited the expression of IL-6, IL-1β, and TNF-α proteins in colonic tissues relative to those in DSS-treated mice that did not receive hordenine (Figure 2D–H).

### 3.3. Hordenine Inhibits S1P/S1PR1/STAT3 Signaling Pathway and Expression of Ras-Related C3 Botulinum Toxin Substrate 1 (Rac1) in Colon Tissues

**Figure 3:**
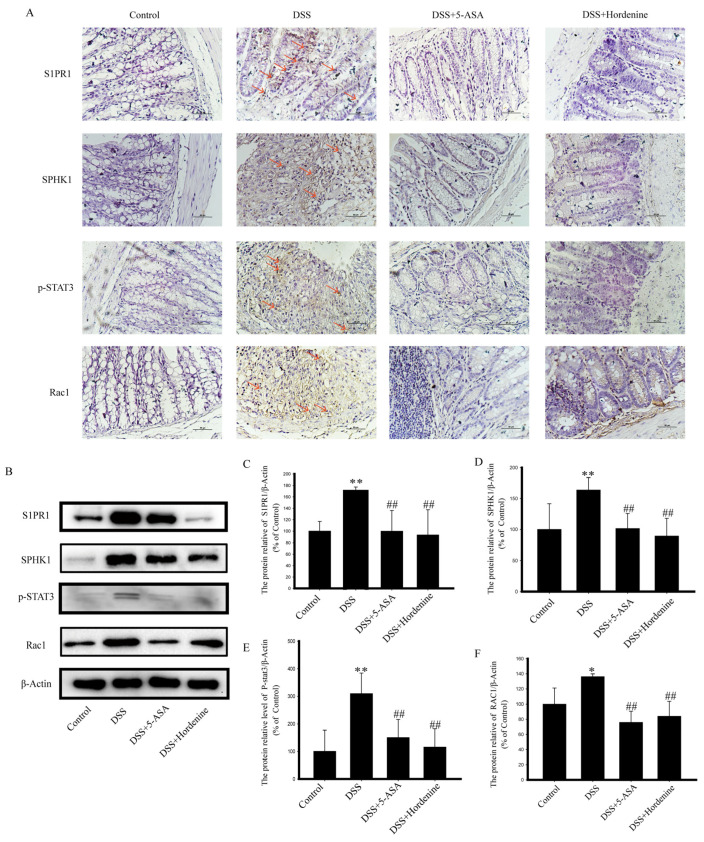
Hordenine (50 mg/kg) inhibited the expression of SPHK1, S1PR1, p-STAT3 and Rac1 in colon tissues. (**A**) Immunohistochemical staining of SPHK1, S1PR1, p-STAT3 and Rac1 in colon tissues. Red arrows point to the positive expression of the target protein, scale bar: 50 μm. (**B**–**F**) Western blotting analysis of SPHK1, S1PR1, p-STAT3 and Rac1 levels in colon tissues. * *p* < 0.05, ** *p* < 0.01 vs. control group; ^##^ *p* < 0.05 vs. DSS group.

As shown in Figure 3A–E, hordenine inhibited DSS-induced increases in the expression of SPHK1 and S1PR1 and the phosphorylation of STAT3 in colon tissues. IHC and western blotting results showed that trends in the expression of Rac1 were consistent with those of SPHK1, S1PR1, and STAT3 (Figure 3A,B,F).

### 3.4. Hordenine Contributes to Mucosal Healing

**Figure 4:**
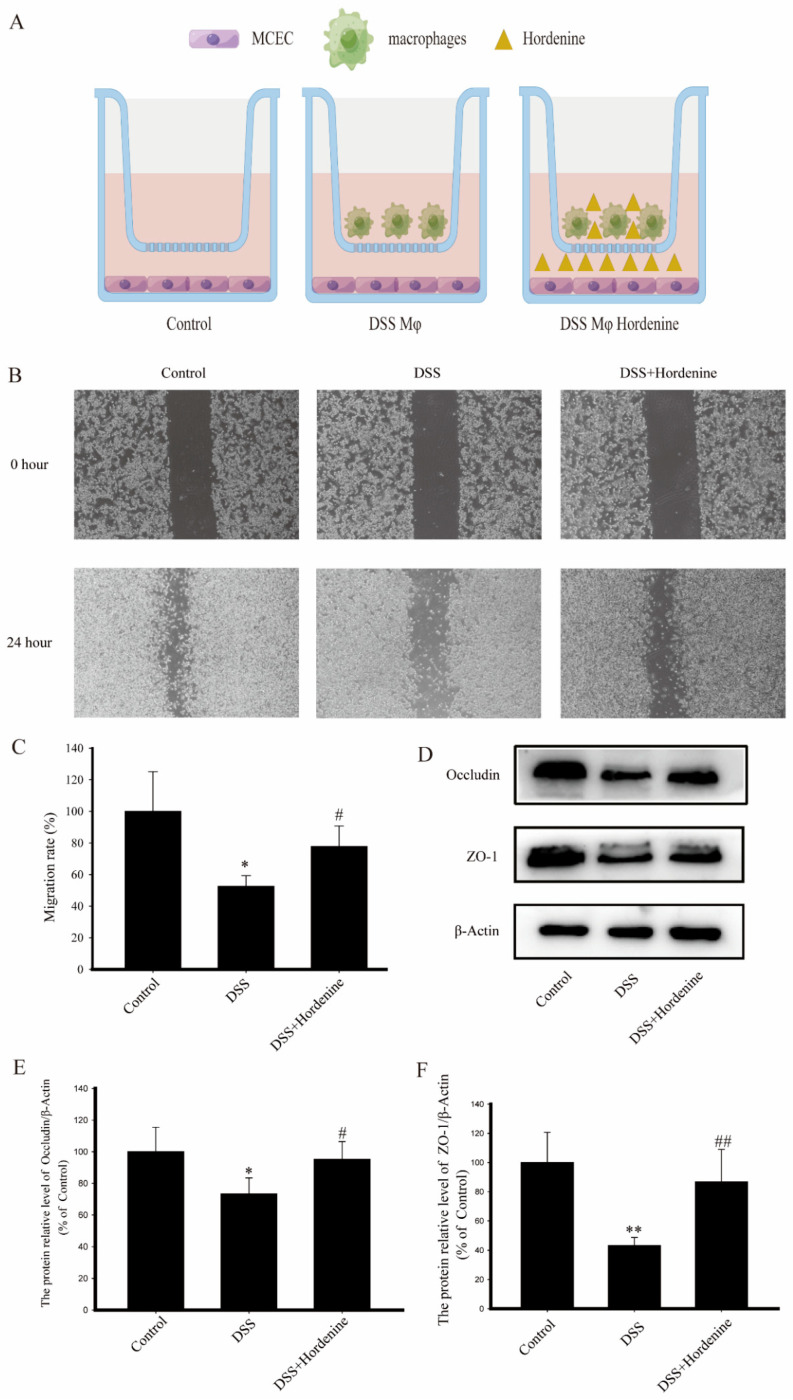
Hordenine (50 mg/kg) affects intestinal epithelial tight junctions. (**A**) Schematic diagram of cell co-culture system. The elements in the graph are derived from figdrow. (**B**,**C**) Scratch experiments showed the migration capacity of MCECs after hordenine treatment. * *p* < 0.05 vs. control group; ^#^ *p* < 0.05 vs. DSS group. (**D**–**F**) Western blotting analysis of occludin and ZO-1 expression in MCECs. * *p* < 0.05, ** *p* < 0.01 vs. control group; ^#^ *p* < 0.05, ^##^ *p* < 0.01 vs. DSS group.

To mimic the inflammatory microenvironment of colon epithelial cells, we established a co-culture of peritoneal macrophages (Mφs) and mouse colonic epithelial cells (MCECs) (Figure 4A). The effects of hordenine on cell migration and wound healing were evaluated using a cell scratch assay. The results indicated that the migration capacity of MCECs decreased in the presence of Mφs from DSS-treated mice, while hordenine-treated Mφs promoted MCECs migration (Figure 4B,C). Western blotting showed that hordenine treatment also significantly increased occludin and ZO-1 expression (Figure 4D–F).

## 4. Discussion

Alkaloid is one of the effective active ingredients in natural Chinese herbal medicine, and is a highly diversified heterocyclic compound [40]. Plant alkaloid is a molecule with anti-inflammatory activity of traditional Chinese medicine, which can inhibit the expression of cytokines, lipid mediators, histamine, enzymes involved in inflammatory reaction and various pro-inflammatory factors [41]. Based on this, alkaloids are important material repositories for drug research and development. As an alkaloid, hordenine is also a promising candidate for the treatment of inflammatory diseases [42]. The DSS-induced UC model has the advantages of being very similar to human clinical symptoms, with a simple operation and high repeatability, and has been commonly used in colitis research [43]. In the present study, we used a DSS-induced UC model to explore the therapeutic effects and mechanisms of hordenine in the treatment of UC. Mice treated with 4% DSS displayed a number of clinical symptoms, including weight loss, diarrhea, and blood in the stool; hordenine treatment substantially improved these symptoms. Histopathological analysis revealed that hordenine markedly improved DSS-induced mucosal necrosis and inflammatory cell infiltration. In addition, the typical tissue changes in the UC mouse model constructed by DSS also include the reduction of goblet cell damage [43]. Our results show that hordenine can significantly inhibit the growth of goblet cells.

Inflammatory cytokines play an important role in the occurrence and development of UC [44], and our results showed that hordenine reversed the DSS-induced increase in the levels of IL-6, IL-1β, and TNF-α, suggesting that hordenine inhibited the typical inflammatory response associated with UC.

Mφs are antigen-presenting cells that connect the innate and adaptive immune systems and have high plasticity [45]. The M1 phenotype of macrophages can up-regulate inflammatory factors and chemokines, promote the production of reactive oxygen species and reactive nitrogen, while the M2 phenotype can inhibit inflammatory reactions and promote wound healing [46]. Inhibiting the polarization of M1 macrophages contributes to reducing DSS-induced colitis damage [47]. In addition, Mφs can drive the immune function of intestinal microenvironment [48]. Changes in the inflammatory microenvironment of the colon epithelium may affect the integrity of the intestinal barrier [44]. To investigate the effects of hordenine on ulcer healing ability in UC mice, Mφs were isolated from UC mice and co-cultured with MCECs in vitro to simulate the inflammatory environment of intestinal epithelial cells. The DSS+Mφs+hordenine group was treated with hordenine (500 µM) on the basis of the DSS+Mφs group, and a blank control group of MCECs was not co-cultured with Mφs. The results of the scratch experiments showed that Mφs isolated from UC mice inhibited the migration of MCEC, whereas hordenine promoted it. Therefore, we infer that hordenine may inhibit the polarization of M1 macrophages or promote the polarization of M2 macrophages to reduce the inflammatory mediators in mice induced by DSS and promote mucosal healing.

The intestinal barrier is a physical barrier formed by various intestinal epithelial cells and cell tight junction complexes, among which are important transmembrane proteins involved in tight junction formation, including occludin and ZO-1 [49]. Studies have shown that the up-regulation of the expression of occludin and ZO-1 can reduce the inflammatory response, improve the tight junctions of the intestinal epithelium, and repair and maintain the intestinal epithelial barrier function [50]. Treatment with hordenine upregulated the expression of occludin and ZO-1, indicating that hordenine can promote colon healing in UC mice. In conclusion, these experiments suggest that hordenine exerts therapeutic effects in UC colon tissue by improving the intestinal barrier via regulation of the expression of tight junction proteins.

**Figure 5:**
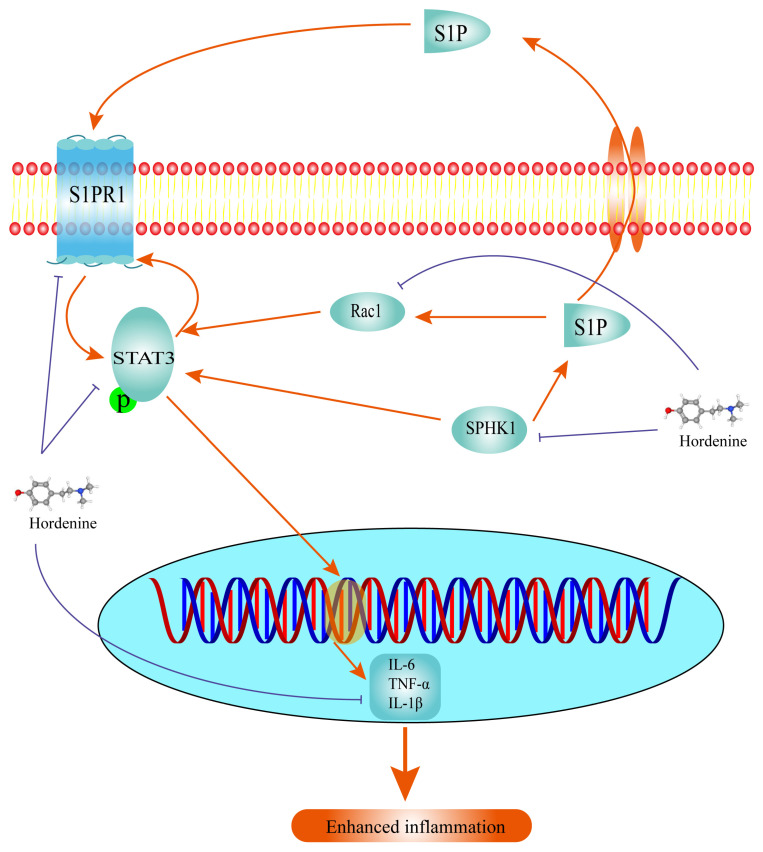
Sphingosine kinase 1 (SPHK1)/sphingosine-1-phosphate receptor 1 (S1PR1)/signal transducer and activator of transcription 3 (STAT3) signaling pathway and the role of ras-related C3 botulinum toxin substrate 1 (Rac1) in hordenine’s inhibition of dextran sodium sulfate-induced ulcerative colitis.

SPHK1 expression is typically low in normal colon tissue. When inflammation occurs, SPHK1 is activated, and its expression is up-regulated to produce inflammation, which also increases in the colon of UC mice [51,52]. S1P plays a key role in inflammatory diseases, especially IBD [53]. STAT3 affects various cytokines and growth factors including IL-6, interferon, epidermal growth factor through phosphorylation [22,54]. Increased expression of p-STAT3 is directly linked to inflammation and the formation of histological lesions. In addition, phosphorylated STAT3 can activate the S1P-SPHK1-S1PR1 signaling axis, which in turn maintains the activated state of STAT3 [55,56]. This process plays an important role in the development of intestinal inflammation [57,58]. Rac1, a member of the Rho family of small GTPases, which are ubiquitously expressed signaling sensors [59,60]. All three receptors of S1P, S1PR1, S1PR2 and S1PR3 can influence the expression of Rac1 [11]. Rac1 activation is necessary for proliferation, migration, and other processes in S1P-mediated cells [50,51,59,60]. Studies have confirmed that Rac1 induces STAT3 activation through the expression and activity of IL-6 to promote inflammation [61]. Our results showed that the expression levels of S1PR1, SPHK1, and their downstream protein p-STAT3 were notably elevated in DSS-treated mice, but were markedly reduced after the administration of hordenine. The expression of Rac1 exhibited a similar trend, suggesting that Rac1 may play a key role in this pathway. As such, we suggest that hordenine exerts therapeutic effects in UC colon tissue through inhibition of the S1P/S1PR1/STAT3 pathway, with its mechanism of action detailed in Figure 5. However, the role of Rac1 in the S1P/S1PR1/STAT3 pathway is not yet comprehensively understood. Future studies should use siRNA and Rac1 overexpression plasmids to explore whether Rac1 plays a bridging role in this pathway. The question of whether hordenine exerts its therapeutic effect on UC by altering the subtype of macrophages also warrants further investigation.

## 5. Conclusions

Our results showed that the administration of hordenine alleviated lesions in DSS-induced UC mice by reducing the expression of pro-inflammatory cytokines and regulating the S1P/S1PR1/STAT3 signaling pathway. Hordenine also promoted the healing of colonic ulcers by regulating the expression of tight junction proteins, including ZO-1 and occludin. The results of this study suggest that hordenine is a promising drug for the treatment of UC.

## Figures and Tables

**Table 1:** The calculation method of disease activity index of all groups.

Score	Weight Loss (%)	Stool Consistency	Fecal Occult Blood
0	0	Normal	Feminine
1	1–5	-	Light blue
2	5–10	Loose Stool	Blue
3	10–15	Mucoid stool	Dark blue
4	>15	Diarrhea	Gross Blood

**Table 2:** Scoring criteria for histological changes.

Score	Number of Ulcers	Epithelial Changes	Lesion Depth
0	0	Normal	Normal
1	1	Goblet cell loss	Mucous membrane
2	2	Massive goblet cell loss	Submucosa
3	3	crypt deletion	Muscle
4	4	Extensive deletion of crypts	Serosa

## Data Availability

Data is contained within the article or supplementary material.

## Supplementary Materials

The following supporting information can be downloaded at: https://www.mdpi.com/article/10.3390/molecules28062834/s1, Table S1: The calculation method of disease activity index of all groups; Table S2: Intestinal lesions assessment criteria.
