# Correction: Xu et al. Beneficial Effects of Hordenine on a Model of Ulcerative Colitis. *Molecules* 2023, *28*, 2834

**DOI:** 10.3390/molecules31132254

**Published:** 2026-06-26

**Authors:** Zhengguang Xu, Qilian Zhang, Ce Ding, Feifei Wen, Fang Sun, Yanzhan Liu, Chunxue Tao, Jing Yao

**Affiliations:** 1 School of Basic Medicine, Jining Medical University, Jining 272067, Chinazhangqilian_11@163.com (Q.Z.); dingceqd@163.com (C.D.); wfei0811@163.com (F.W.); sunfangfangsun@126.com (F.S.); liuyanzhan8098@163.com (Y.L.); taoxue1231222@163.com (C.T.); 2 School of Basic Medicine, Weifang Medical University, Weifang 261000, China; 3 Jining Key Laboratory of Pharmacology, Jining Medical University, Jining 272067, China

Figure Legend

In the original publication [1], there was a mistake in the legend for Figure 4B,C. The original figure legend did not describe how the representative scratch images were selected. The correct legend of Figure 4B,C appears below.

Scratch experiments showed the migration capacity of MCECs after hordenine treatment. * *p* < 0.05 vs. control group; # *p* < 0.05 vs. DSS group. The images shown are from a representative experiment with wound closure near the average of three independent experiments.

Error in Figure

In the original publication, there was a mistake in Figure 4 as published. In Figure 4B, the 24 h image for the DSS group was incorrectly placed due to file name confusion (this error is unrelated to the quantitative data), and the 24 h image for the DSS + Hordenine group was selected from the single experiment with the highest effect, which does not accurately reflect the mean of three independent experiments presented in the bar graph. The corrected Figure 4 appears below.

The authors state that the scientific conclusions are unaffected. This correction was approved by the Academic Editor. The original publication has also been updated.

**Figure 4:**
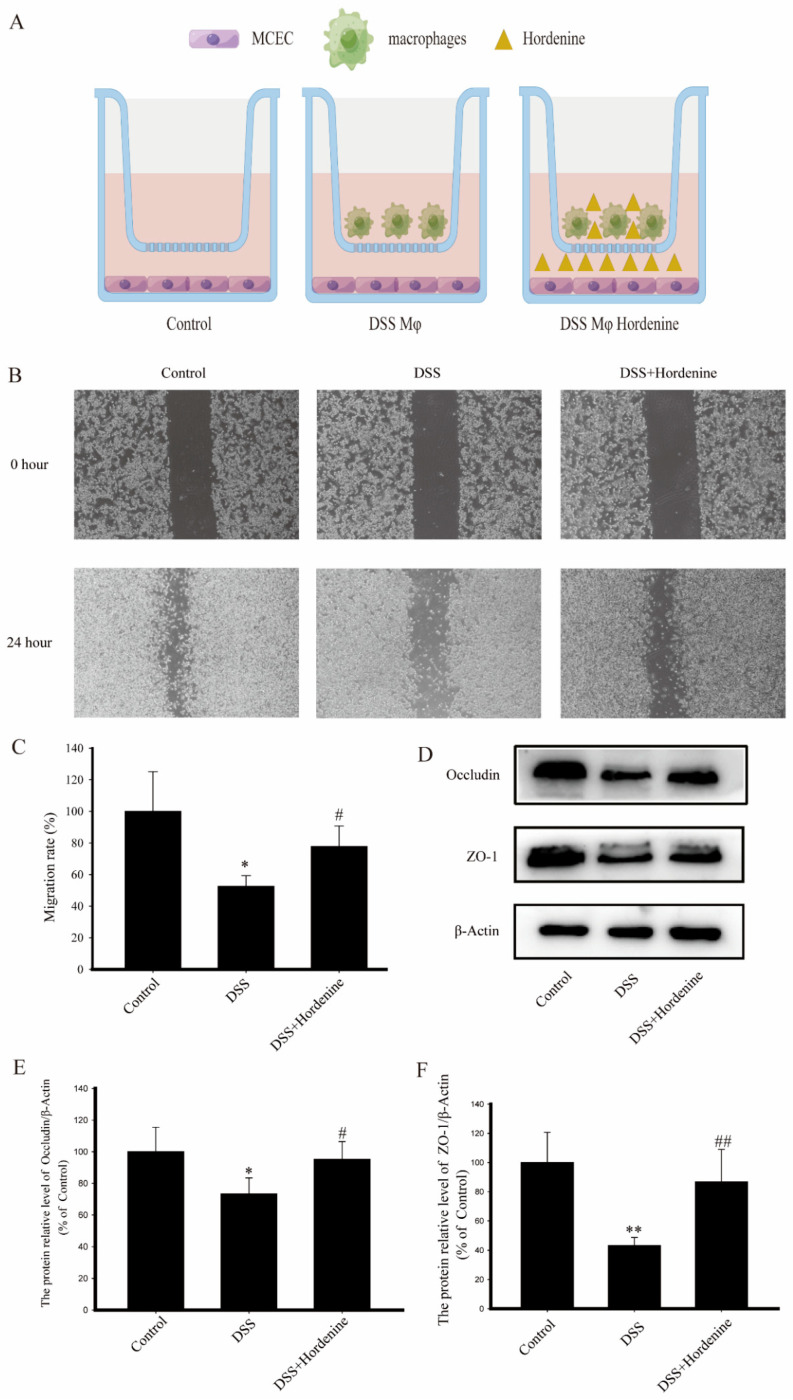
Hordenine (50 mg/kg) affects intestinal epithelial tight junctions. (**A**) Schematic diagram of cell co-culture system. The elements in the graph are derived from figdrow. (**B**,**C**) Scratch experiments showed the migration capacity of MCECs after hordenine treatment. * *p* < 0.05 vs. control group; # *p* < 0.05 vs. DSS group. The images shown are from a representative experiment with wound closure near the average of three independent experiments. (**D**–**F**) Western blotting analysis of occludin and ZO-1 expression in MCECs. * *p* < 0.05, ** *p* < 0.01 vs. control group; # *p* < 0.05, ## *p* < 0.01 vs. DSS group.
