# Supplementary material for: Beneficial Effects of Hordenine on a Model of Ulcerative Colitis
Source: Molecules. 2023 Mar 21;28(6):2834. doi: 10.3390/molecules28062834 (PMC10054341; doi:10.3390/molecules28062834)
Supplement: Supplementary file 1 [file molecules-28-02834-s001.zip › molecules-2172042-supplementary.pdf]

Supplementary Material

Beneficial Effects of Hordenine on a Model of Ulcerative Colitis

Zhengguang Xu [1,†], Qilian Zhang [1,2,†], Ce Ding [1,†], Feifei Wen [1], Fang Sun [1,3], Yanzhan Liu [1], Chunxue Tao [1] and Jing Yao [1,3,*]

[1] School of Basic Medicine, Jining Medical University, Jining 272067, China
[2] School of Basic Medicine, Weifang Medical University, Weifang 261000, China
[3] Jining Key Laboratory of Pharmacology, Jining Medical University, Jining 272067, China
* Correspondence: yjing_87@163.com
† These authors contribute equally to this work.

Table S1. The calculation method of disease activity index of all groups.

Score	Weight Loss	Stool Consistency	Fecal Occult Blood
0	0	Normal	Feminine
1	1-5%	——	Light blue
2	5-10%	Loose stool	Blue
3	10-15%	Mucoid stool	Dark blue
4	＞15%	Diarrhea	Gross Blood

(Fecal occult blood was detected by fecal occult blood qualitative detection kit purchased from Shanghai Yuanye Bio-Technology Co., Ltd. (Shanghai, China)).

Table S2. Intestinal lesions assessment criteria.

Score	Number of Ulcers	Epithelial Changes	Lesion Depth
0	0	Normal	Normal
1	1	Goblet cell loss	Mucous membrane
2	2	Massive goblet cell loss	Submucosa
3	3	crypt deletion	Muscle
4	4	Extensive deletion of crypts	Serosa
